# Atrial natriuretic factor: is it responsible for hyponatremia and natriuresis in neurosurgery?

**DOI:** 10.5935/0103-507X.20160030

**Published:** 2016

**Authors:** Ana Paula Devite Cardoso Gasparotto, Antonio Luis Eiras Falcão, Carolina Kosour, Sebastião Araújo, Eliane Araújo Cintra, Rosmari Aparecida Rosa Almeida de Oliveira, Luiz Claudio Martins, Desanka Dragosavac

**Affiliations:** 1 Department of Surgery, Faculdade de Medicina, Universidade Estadual de Campinas - Campinas (SP), Brazil.; 2 Department of Nursing, Faculdade de Medicina, Universidade Estadual de Campinas - Campinas (SP), Brazil.; 3 Department of Physiotherapy, Pontifícia Universidade Católica de Campinas - Campinas (SP), Brazil.

**Keywords:** Sodium, Hyponatremia, Natriuresis, Atrial natriuretic factor, Neurosurgery

## Abstract

**Objective:**

To evaluate the presence of hyponatremia and natriuresis and their association with atrial natriuretic factor in neurosurgery patients.

**Methods:**

The study included 30 patients who had been submitted to intracranial tumor resection and cerebral aneurism clipping. Both plasma and urinary sodium and plasma atrial natriuretic factor were measured during the preoperative and postoperative time periods.

**Results:**

Hyponatremia was present in 63.33% of the patients, particularly on the first postoperative day. Natriuresis was present in 93.33% of the patients, particularly on the second postoperative day. Plasma atrial natriuretic factor was increased in 92.60% of the patients in at least one of the postoperative days; however, there was no statistically significant association between the atrial natriuretic factor and plasma sodium and between the atrial natriuretic factor and urinary sodium.

**Conclusion:**

Hyponatremia and natriuresis were present in most patients after neurosurgery; however, the atrial natriuretic factor cannot be considered to be directly responsible for these alterations in neurosurgery patients. Other natriuretic factors are likely to be involved.

## Abstract (pt)

**Objetivo:**

Avaliar a presença de hiponatremia e natriurese, bem como suas associações com o fator natriurético atrial em pacientes de neurocirurgia.

**Métodos:**

Foram incluídos 30 pacientes submetidos à ressecção de tumor intracraniano e à clipagem de aneurisma cerebral. Os níveis plasmáticos e urinários de fator natriurético atrial foram medidos durante os períodos pré e pós-operatório.

**Resultados:**

Hiponatremia esteve presente em 63,33% dos pacientes, particularmente no primeiro dia pós-operatório. Observou-se natriurese em 93,33% dos pacientes, principalmente no segundo dia pós-operatório. Os níveis plasmáticos de fator natriurético atrial estavam aumentados em 92,60% dos pacientes em pelo menos um dos dias pós-operatórios, mas não houve associação estatisticamente significante entre fator natriurético atrial e sódio plasmático, e entre fator natriurético atrial e sódio urinário.

**Conclusão:**

Após neurocirurgia, na maior parte dos pacientes, estiveram presentes hiponatremia e natriurese; contudo, o fator natriurético atrial não pôde ser considerado diretamente responsável por tais alterações nos pacientes neurocirúrgicos. Provavelmente, há o envolvimento de outros fatores natriuréticos.

## INTRODUCTION

Disorders of plasma sodium concentration expose cells to hypotonic or hypertonic stress. Although all cells are affected, the clinical manifestations are primarily neurologic. Rapid changes in plasma sodium concentrations can cause severe, permanent and sometimes lethal brain injury. Those disorders are common in neurology and neurosurgery patients who already have cerebral edema from the primary injury and whose adaptive mechanisms may be impaired with the worsening of the patients' neurologic condition.^(1-7)^

Hyponatremia is defined as a serum sodium concentration < 135mEq/L.^(2)^ It is the most commonly found sodium disturbance in neurosurgery patients. Hyponatremia is usually associated with natriuresis (sodium renal loss > 20mEq/L) and worse neurological states. Cerebral salt wasting syndrome (CSWS) is defined as the renal loss of sodium during intracranial disease, which leads to hyponatremia. A decrease in extracellular fluid volume is the main cause of these alterations in neurosurgery patients.^(1-4,8-22)^

The mechanism by which intracranial disease leads to renal salt wasting is not completely understood. The regulation of sodium homeostasis involves both humoral and neural mechanisms. Humoral factors include the renin-aldosterone axis, atrial natriuretic factor, and antidiuretic hormone. Neural factors include the direct neural modulation of tubular sodium reabsorption and the indirect neural modulation of rennin release. Natriuretic factors may play an important role in CSWS,^(1,3,4,8,9,11,12,15,19,22)^ and in recent years, several reports have attempted to identify a causal relationship between natriuretic peptide and CSWS. Amongst the various natriuretic factors, atrial natriuretic factor (ANF) might be the most probable candidate to mediate CSWS.^(3,4,8-10,12,13,16,23-25)^

ANF is produced in and released from the atrial appendages, and it seems to act in different tissues, participating in the control of fluid balance with a negative sodium balance and changes in blood volume. ANF has also been identified in areas of the central nervous system involved in cardiovascular, sodium and fluid regulation.^(2,10,12,14,16)^

ANF-containing neurons have been identified in the rat hypothalamus and lamina terminalis; however, the concentration of ANF in the brain is 10000 times lower than in the heart, making it unlikely that the brain secretion of ANF is responsible for CSWS. Although atrial stretch is thought to be the principal mechanism for cardiac ANF release, there is evidence that the central nervous system modulates cardiac ANF secretion. Intracranial disease may lead to a disturbance of the brain's control over ANF secretion, and, under certain conditions, excessive ANF is secreted.^(12)^

The purpose of the present study was to verify the presence of hyponatremia and natriuresis and their relationships with atrial natriuretic factor in neurosurgery patients.

## METHODS

This prospective observational study was performed in an academic teaching hospital. It was approved by the *Faculdade de Ciências Médicas* Ethics Committee of the *Universidade Estadual de Campinas* (UNICAMP) under protocol number 142/99. Signed informed consent was obtained from the patients or family members prior to inclusion in the study.

Thirty consecutive male and female patients older than 13 years of age who had been submitted to elective neurosurgery either for the resection of a brain tumor or clipping of an aneurism of the cerebral artery with or without subarachnoid hemorrhage (SAH) were enrolled in the study. No patients had a recent history of head trauma, diagnosis of pituitary tumor, age ≤ 13 years, pregnancy or alterations in cardiac, renal, adrenal, thyroid or hepatic function. Postoperative fluid and sodium administration were always > 2L/day of normal saline solution and were adjusted to maintain a normal intravascular volume and to avoid a negative sodium balance.

The patient's age was recorded and the preoperative patient's neurological status was assessed with the Glasgow Coma Scale.

Urinary samples were collected over a period of 12 hours overnight and cooled immediately to 4°C until the end of the collection period on the day before surgery (D0) and on the first to fifth postoperative days (D1 - D5). The urinary sodium concentration was determined by indirect potentiometry.

Blood samples were collected from a previously placed venous catheter.

Plasma sodium concentrations were determined each morning on the preoperative day (D0) and on the first to fifth postoperative days (D1 - D5) by the selective ion technique.

Plasma ANF concentrations were determined on the preoperative day (D0) and on postoperative days 1, 3 and 5 (D1, D3, D5). Blood samples were collected in tubes containing ethylenediamine tetraacetic acid - EDTA (1mg/mL), cooled to approximately +4^o^C, and centrifuged at 3000 rpm for 15 minutes. Plasma was frozen (-20°C) and sent to the laboratory on dry ice for peptide assay.^(1,26,27)^ Atrial natriuretic factor was measured in duplicate by radioimmunoassay after the acetone-ether extraction of plasma.

Natriuresis was defined as urinary sodium higher than 110mEq/12 hours (normal urinary sodium is 20 - 110mEq/12 hours).

Hyponatremia was defined as a plasma sodium concentration below 135mEq/L (normal plasma sodium is 135 - 146mEq/L) at least once in the clinical course.

High plasma ANF levels were defined as a plasma ANF higher than 50pg/mL (normal is 25 - 50pg/mL).

### Statistical analysis

Descriptive statistics of numeric variables were performed to describe the profile of the sample. The results were expressed as the mean (± standard deviations - SD). Analysis of variance for repeated measures was used to compare longitudinal measures among time (repeated measures ANOVA). Comparative analysis between plasma ANF and plasma sodium and between plasma ANF and urinary sodium was studied by Fisher's exact test. We also studied the correlation between variations in plasma ANF and plasma sodium and between variations in plasma ANF and urinary sodium on the first postoperative day by Spearman's correlation coefficient. Statistical significance was defined as p < 0.05.

## RESULTS

The study included 30 patients, of which 19 were diagnosed with intracranial tumor (63.33%) and 11 with cerebral artery aneurism (36.7%).

Their mean age was mean 44 ± 18 and the preoperative Glasgow Coma Score ranged from 10 to 15 (mean 13 ± 2).

Plasma sodium, urinary sodium and plasma ANF are presented in table 1 and described below.

**Table 1:** Numeric variables

**Variable**	**N**	**Mean ± SD**
Naplasm 0	30	135.37 ± 3.80
Naplasm 1	30	135.47 ± 5.24
Naplasm 2	29	136.21 ± 3.80
Naplasm 3	30	136.60 ± 4.72
Naplasm 4	25	136.04 ± 4.75
Naplasm 5	20	136.20 ± 4.32
ANF 0	26	83.49 ± 55.54
ANF 1	26	103.22 ± 75.93
ANF 3	26	108.79 ± 60.86
ANF 5	20	56.60 ± 45.63
Nauri 0	26	119.62 ± 120.91
Nauri 1	25	243.72 ± 141.15
Nauri 2	28	269.57 ± 157.39
Nauri 3	27	169.48 ± 113.54
Nauri 4	26	183.55 ± 122.06
Nauri 5	17	220.71 ± 184.02

SD - standard deviation; Naplasm 0 - plasma sodium on the preoperative day; Naplasm 1 - Naplasm 5 - plasma sodium on the first to fifth postoperative days; ANF 0 - atrial natriuretic factor on the preoperative day; ANF 1 - ANF 5 - atrial natriuretic factor on the first to fifth postoperative days; Nauri 0 - urinary sodium on the preoperative day; Nauri 1 - Nauri 5 - urinary sodium on the first to fifth postoperative days.

### Urinary sodium

**Figure 1:**
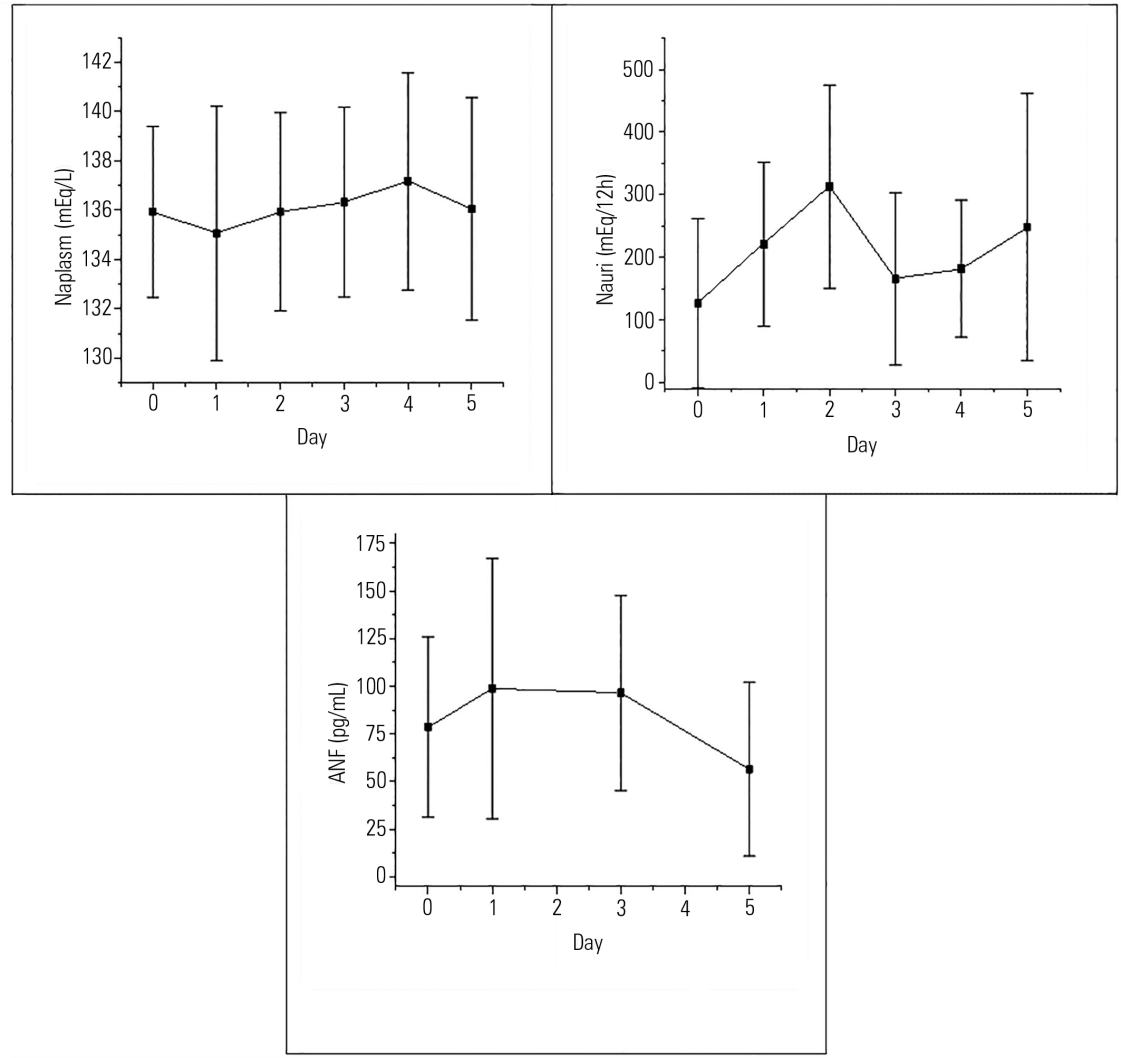
The average and standard deviation of plasma sodium (mEq/L), urinary sodium (mEq/12 hours) and plasma atrial natriuretic factor (pg/mL) in the pre- and postoperative time periods (D0 and D1 - 5). ANF - atrial natriuretic factor; Naplasm - plasma sodium; Nauri - urinary sodium.

Natriuresis (urinary sodium > 110mEq/12 hours) was observed in 28 of 30 patients (93.33%) at least in one day during the postoperative period (D1 - D5). Higher levels were found on D2 (269.57 ± 157.39mEq/12 hours), when 85.71% of the patients presented natriuresis (Table 1 and Figure 1) with a statistically significant difference (p = 0.012).

### Plasma sodium

Hyponatremia (plasma sodium ≤ 135mEq/L) was present in 19 of 30 patients (63.33%) in the postoperative period (D1 - D5), and the lowest plasma sodium levels were found on D1 (135.47 ± 5.24), when 40% of the patients presented hyponatremia (Table 1 and Figure 1). However, there was no statistically significant difference in the plasma sodium levels during this time period (p = 0.726).

### Atrial natriuretic factor

Plasma ANF levels remained increased (> 50pg/mL) on at least one of the postoperative days (D1 - D5) in 92.60% of the patients. Higher levels were found on D3 (108.79 ± 60.86pg/mL) and decreased on D5 (56.60 ± 45.63pg/mL) (Table 1 and Figure 1). There was no statistically significant association between plasma ANF and plasma sodium and between plasma ANF and urinary sodium in any of the days studied (Table 2). When we studied the correlation between plasma ANF and plasma sodium and between plasma ANF and urinary sodium on the first postoperative day, we also did not find any significant correlation (p = 0.3742 and p = 0.3139, respectively).

**Table 2:** Comparative analysis among the levels of atrial natriuretic factor, plasma sodium and urinary sodium at each time point (Fisher’s exact test)

	**ANF ≤ 50pg/mL**	**ANF > 50pg/mL**	**p value**
Naplasm 0			
< 135mEq/L	1	6	0.375
≥ 135mEq/L	7	12	
Naplasm 1			
< 135mEq/L	2	8	0.668
≥ 135mEq/L	5	11	
Naplasm 3			
< 135mEq/L	0	8	0.529
≥ 135mEq/L	3	15	
Naplasm 5			
< 135mEq/L	4	0	0.087
≥ 135mEq/L	6	9	
Nauri 0			
≤ 110mEq/L	4	11	0.630
> 110mEq/L	3	4	
Nauri 1			
≤ 110mEq/L	0	4	0.255
> 110mEq/L	7	10	
Nauri 3			
≤ 110mEq/L	2	5	0.210
> 110mEq/L	1	15	
Nauri 5			
≤ 110mEq/L	2	2	1.000
> 110mEq/L	5	7	

ANF - plasma atrial natriuretic factor on each day; Naplasm 0 - plasma sodium on the preoperative day; Naplasm 1- Naplasm 5 - plasma sodium on the first to fifth postoperative days; Nauri 0 - urinary sodium on the preoperative day; Nauri 1 - Nauri 5 - urinary sodium on the first to fifth postoperative days.

## DISCUSSION

Hyponatremia is the sodium disturbance most frequently found in neurosurgery patients. Although hyponatremia is most reliably encountered in patients with aneurismal SAH, it sometimes occurs in a variety of other conditions that affect the central nervous system, such as malignancy and head trauma; it has also been reported in the postoperative neurosurgical setting.^(11)^ It is frequently associated with natriuresis, and its main cause is CSWS.^(1-4,8-22)^ CSWS is still not completely understood, and natriuretic factors seem to play an important role in its physiopathology. Among the natriuretic factors, ANF may play a role in the hyponatremia and natriuresis found in neurosurgical patients, as shown in previous studies.^(1,3,4,8-13,15,16,19,22-25)^

However, there are conflicting reports regarding ANF in neurologic patients. Some studies have demonstrated that ANF produces natriuresis and diuresis when administered either peripherally or centrally.^(24,25)^ Isotani et al. demonstrated that hyponatremia produced significantly elevated levels of ANF and vasopressin immediately after SAH. They observed that ANF remained high in patients with mild hyponatremia and concluded that ANF may be a causal natriuretic factor in CSWS.^(13)^ Doczi et al. reported that only the SAH patients with elevated intracranial pressure (> 20mmHg) had increased plasma ANF concentrations.^(28)^ A direct relationship between ANF and intracranial pressure was also reported by Berendes et al., which suggested that the development of renal salt wasting is a protective measure that limits extreme rises in intracranial pressure.^(29)^ Weinand et al. found serum ANF levels to be elevated above the normal range in 6 of 8 neurosurgical patients with a variety of neurosurgical disorders, including cerebral tumors. Additionally, a near-linear relationship was observed between plasma ANF levels and urine sodium excretion.^(30)^

Other studies also showed elevated concentrations of circulating ANF in neurosurgery patients and after SAH, but no clear relationship with hyponatremia and natriuresis has been established.^(2,9,12,13,31)^ In a study with 25 patients with SAH, Diringer et al. observed that ANF levels were significantly elevated in 21 patients with SAH compared with 4 unruptured aneurysms and returned to normal over 2 weeks. There was no correlation between ANF and serum sodium levels, and the ANF levels in 2 patients with SAH who had hyponatremia were not significantly different from those in the other patients with SAH. Thus, elevated levels of ANF alone do not account for the hyponatremia observed after SAH.^(2)^ Another study prospectively studied sodium, volume regulation and ANF in 19 patients following acute aneurismal SAH. Plasma ANF values were elevated but did not correlate with the presence of hyponatremia.^(31)^ Elevated levels of ANF following SAH were also demonstrated by Diringer et al. (1991) in another study and they may represent a marker of hypothalamic dysfunction but may not directly contribute to hyponatremia themselves.^(32)^

In a prospective study of 49 patients with SAH, Tsubokawa et al. observed that the plasma ANF concentrations were not altered.^(33)^ Normal ANF plasma levels have been found in patients with CSWS associated with parietal glioma^(11)^ and after surgery for pituitary adenoma.^(34)^ During experimental natriuresis induced by the intracerebroventricular administration of hypertonic saline, plasma ANF levels were found to decrease.^(35)^

In our study, hyponatremia (plasma sodium < 135mEq/L) was found in 63.33% of the patients in the postoperative phase, particularly on the first postoperative day. Natriuresis (urinary sodium > 110mEq/12 hours) was observed in 93.33% of the patients. Urinary sodium levels increased during the entire postoperative period, particularly on the second postoperative day; however, their levels were also high in the preoperative period. We also observed high plasma ANF levels during the entire postoperative period; however, high plasma ANF levels were also found in the preoperative period, similar to urinary sodium, likely due to the primary disease. Although hyponatremia, natriuresis and increased plasma ANF levels were found in the postoperative period, we did not find any significant statistical correlation among them, which suggested that ANF is not responsible for hyponatremia and natriuresis in neurosurgery patients.

Our observations confirm the previous reports of some authors.^(2,31,32)^ We found high plasma ANF levels without a correlation with hyponatremia and natriuresis, as shown by Diringer et al.^(2,31,32)^ Tsubokawa et al.,^(33)^ Yamaki et al.^(34)^ and Hansel et al.^(35)^ These authors also did not find any correlation with ANF, hyponatremia and natriuresis; however, they did not find high plasma ANF levels, as we observed in our study.

The limitations of the present study include the small number of patients studied; however, studies involving CSWS and ANF in the literature are mostly review articles, case reports and retrospective studies involving small numbers of patients. Urinary sodium was measured at 12 hours overnight instead of 24 hours, and plasma ANF was measured only on the preoperative day and the first, third and fifth postoperative days. Further experimental studies in these areas must be performed, including the investigation of other natriuretic factors.

## CONCLUSION

Hyponatremia and natriuresis are commonly found in neurosurgical patients. Despite increased plasma concentrations of atrial natriuretic factor in the postoperative period, it is not directly responsible for hyponatremia and natriuresis. The presence of other natriuretic factors, the participation of multiple natriuretic factors alone or in combination, and direct neural effects on the kidneys may also be involved.
